# Optimal adaptive heuristic algorithm based energy optimization with flexible loads using demand response in smart grid

**DOI:** 10.1371/journal.pone.0307228

**Published:** 2024-11-05

**Authors:** Hisham Alghamdi, Lyu-Guang Hua, Ghulam Hafeez, Sadia Murawwat, Imen Bouazzi, Baheej Alghamdi

**Affiliations:** 1 Department of Electrical Engineering, College of Engineering, Najran University, Najran, Saudi Arabia; 2 Power China Hua Dong Engineering Corporation Limited, Hangzhou, China; 3 Department of Electrical Engineering, University of Engineering and Technology, Mardan, Pakistan; 4 Department of Electrical Engineering, Lahore College for Women University, Lahore, Pakistan; 5 Department of Industrial Engineering, College of Engineering, King Khalid University, Abha, Saudi Arabia; 6 Center for Engineering and Technology Innovations, King Khalid University, Abha, Saudi Arabia; 7 Smart Grids Research Group, Center of Research Excellence in Renewable Energy and Power Systems, King Abdulaziz University, Jeddah, Saudi Arabia; 8 Department of Electrical and Computer Engineering, Faculty of Engineering, King Abdulaziz University, Jeddah, Saudi Arabia; Università di Pisa, ITALY

## Abstract

Demand response-based load scheduling in smart power grids is currently one of the most important topics in energy optimization. There are several benefits to utility companies and their customers from this strategy. The main goal of this work is to employ a load scheduling controller (LSC) to model and solve the scheduling issue for household appliances. The LSC offers a solution to the primary problems faced during implementing demand response. The goal is to minimize peak-to-average demand ratios (PADR) and electricity bills while preserving customer satisfaction. Time-varying pricing, intermittent renewable energy, domestic appliance energy demand, storage battery, and grid constraints are all incorporated into the model. The optimal adaptive wind-driven optimization (OAWDO) method is a stochastic optimization technique designed to manage supply, demand, and power price uncertainties. LSC creates the ideal schedule for home appliance running periods using the OAWDO algorithm. This guarantees that every appliance runs as economically as possible on its own. Most appliances run the risk of functioning during low-price hours if just the real time-varying price system is used, which could result in rebound peaks. We combine an inclined block tariff with a real-time-varying price to alleviate this problem. MATLAB is used to do a load scheduling simulation for home appliances based on the OAWDO algorithm. By contrasting it with other algorithms, including the genetic algorithm (GA), the whale optimization algorithm (WOA), the fire-fly optimization algorithm (FFOA), and the wind-driven optimization (WDO) algorithms, the effectiveness of the OAWDO technique is supported. Results indicate that OAWDO works better than current algorithms in terms of reducing power costs, PADR, and rebound peak formation without sacrificing user comfort.

## Introduction

In recent years, the surging demand for energy and the compelling need to address environmental concerns have propelled renewable energy sources (RESs) and demand response (DR) to the forefront of sustainable energy solutions [1]. The emergence of smart power grids (SPG) accommodates RESs and DR to meet consumer demand with low pollution emissions [2]. Despite this potential, the optimal operation of these hybrid renewable sources and the active engagement of consumers in DR pose complex challenges that necessitate innovative control, management and energy/load scheduling strategies [3]. Also, with the use of RESs the problem of voltage and frequency stabilization emerged [4, 5]. This imperative directs the focus of this research toward unlocking the full potential of renewable resources and DR while ensuring economic viability and grid stability. With a specific emphasis on optimal energy optimization considering DR and RESs, this study aims to develop novel methodologies that seamlessly incorporate RESs and address energy optimization through load scheduling under DR. By tackling factors such as system constraints, consumer constraints, demand fluctuations, and the integration of energy storage systems (ESS)/ electric vehicles (EVs) [6–8] this research endeavors to formulate efficient energy optimization models that strike a balance among supply, demand, and storage elements. The anticipated outcomes of this research hold promise for advancing energy optimization within the context of SPG. This advancement is expected to bolster energy security, minimize energy costs, relieve the load burden on the main grid, and pave the way for more sustainable and resilient energy ecosystems [9].

To solve energy optimization problems using DR and load scheduling, the related works are grouped into five main families based on the approaches: mathematical, game theoretic, machine learning, heuristic, and meta-heuristic. Each family is elaborated as follows.

Mathematical techniques, also referred to as deterministic techniques, encompass methodologies such as integer linear programming (ILP), mixed integer linear programming (MILP), non-linear programming (NIP), and mixed integer non-linear programming (MINLP) [10]. For example, ILP and binary ILP mechanisms for managing household load through consumption scheduling in the SPG. The optimal and balanced daily load schedule aims to minimize the peak hourly load [11, 12]. However, the load uncertainty persists. The authors introduced a MILP approach-based framework for addressing the energy management problems within microgrids [13]. The primary goal of the energy management problem is to establish an optimal generation and controllable load demand strategy over a specified planning horizon. This strategy aims to minimize operational costs while adhering to both economic and technical constraints. However, the modeling of microgrid reliability and uncertainty still requires attention. A novel optimal scheduling model utilizing MILP is formulated for the robust and optimal scheduling of isolated microgrids [14]. This novel method successfully addresses energy optimization by integrating multiple DR and a green hydrogen-based storage technology [15]. The authors formulated an MILP model for optimizing the energy efficiency of multi-energy power grid systems in stadiums. Experimental results demonstrated that the optimized power grid system, employing MILP, led to a reduction in the total monthly power supply cost, a maximum decrease in pollution, and an average reduction in the working time of the power supply system [16]. The application of MILP is employed for scheduling smart home energy sources and appliances, taking into account users’ weekly and daily plans to meet the preferences of home occupants. The proposed approach achieves a 20% reduction in fuel consumption while maintaining a relatively comfortable lifestyle for home users [17]. An optimization framework based on NLP is implemented for the automated scheduling of tasks and energy resources in smart homes. The objective is to fulfill the user’s electrical and thermal comfort requirements collectively [18]. An MINLP approach is devised to address the energy disaggregation problem. The proposed formulation demonstrated computational efficiency, effectively distinguishing between loads with comparable consumption patterns. Moreover, it successfully reconstructed the signatures of known appliances, even in the presence of unmetered device [19]. An optimal scheduling methodology utilizing the MILP is employed for energy systems integrated into buildings. The devised scheduling strategy aims to minimize the overall day-ahead operational cost, encompassing both energy usage costs and penalties associated with plant on/off operations [20]. The mathematical methods, including ILP, MILP, NLP, and MINLP, face challenges such as computational complexity, issues related to solution quality, linearization errors, sensitivity to input data accuracy, model complexity, lack of robustness, and difficulty in handling uncertainties associated with load/generation while addressing energy optimization problems in SPG.

To address the limitations of mathematical methods, researchers and practitioners have explored game-theoretic approaches to energy optimization problems. For example, the authors used dynamic pricing schemes to schedule the SPG community’s energy consumption using a bi-level game theory approach, which aims to reduce peak demand in the neighborhood [21]. A robust game-theoretic method to balance energy consumption and storage capacity while accounting for distributed generation’s unpredictability. Through the incorporation of aspects including distributed generation costs, energy storage costs, and bidirectional energy trading, the study investigates energy management and storage optimization [22]. A model utilizing game theory is employed to tackle the energy management issue outlined in [23], incorporating the presence of electric vehicles in the SPG. In [24], a model based on game theory is formulated to tackle the demand-side management issue related to thermostatically controlled loads, incorporating an innovative pricing mechanism. Similarly, another game-theoretic model is established, encompassing five loads and pricing datasets, to handle power scheduling across various time spans, including regular periods, public holidays, weekends, vacations, etc. [25]. A hierarchical control methodology employing a Stackelberg game-theoretic model is implemented for load regulation, taking into account uncertainties and randomness in [26]. The objective is to facilitate interaction between the distribution system operator (leader) and load aggregator (follower) within the electricity market, with the aim of maximizing utility through a compensation pricing scheme. Game-theoretic models demonstrate effectiveness in addressing energy optimization problems. However, they face limitations, such as restricted applicability, complexity, non-uniqueness of solutions, reliance on accurate input data, and implementation challenges.

Hence, heuristic and meta-heuristic optimization approaches have emerged to tackle challenges associated with game-theoretic methodologies [27–29]. For example, the authors employed the genetic algorithm to address the energy optimization problem, aiming to reduce energy costs and electricity bills through residential loads scheduling [30–34]. Further, distributed algorithms are developed to solve economic dispatch problems [35, 36]. Researchers have devised evolutionary algorithms to tackle energy optimization challenges through load scheduling [37–39]. The PSO algorithm-based energy management model is developed to optimize costs and emissions in a microgrid with energy storage [40]. However, this approach addresses only the cost and emissions while neglecting other interrelated objectives such as PADR and comfort. Likewise, in [41], PSO is applied for DSM to address the charging/discharging scheduling of storage batteries. The objective is to decrease the peak power demands and avoid batteries overcharging/over-discharging while ensuring minimal thermal discomfort. Additionally, a hybrid algorithm of glow-worm swarm optimization and support vector machine is developed to tackle the load rescheduling problem, aiming to minimize end-user costs in [42]. Heuristic and meta-heuristic algorithms prove highly effective in addressing load scheduling problems as they can efficiently manage stochastic problems, delivering almost the best solutions within a practical timeframe. Nevertheless, it’s important to note that these algorithms cannot guarantee the identification of the best solution, as their effectiveness relies on parameter tuning and the nature of primary solutions.

The authors developed a machine learning model to address energy optimization problems in SPG [43]. A hybrid approach, including reinforcement learning and deep learning, is utilized to optimize schedules for real-time energy management in buildings. This enables the provision of instant feedback to consumers, promoting more efficient electricity usage [44, 45]. Machine learning models for energy optimization in SPGs encounter challenges such as reliance on historical data, sensitivity to outliers, and difficulties in adapting to dynamic environments. Additionally, concerns encompass interpretability issues, limited generalization, and the environmental impact of resource-intensive models. Overcoming these limitations is essential to guarantee the effectiveness and reliability of machine learning applications for energy optimization in SPGs.

To tackle the aforementioned challenges, a novel and adaptive heuristic algorithm, namely OAWDO, is introduced to enhance the performance of the heuristic algorithm WDO. Additionally, the LSC model is introduced to address energy optimization using DR with flexible loads in SPG. Additionally, the LSC model is introduced to address energy optimization using DR with flexible loads in SPG. Prior research has developed a comprehensive understanding of energy optimization and put forth an energy optimization approach that centers around reducing electricity expenses using flexible load scheduling and distributed resources. This singular emphasis on electricity bills often leads to the creation of rebound peaks and discomfort for consumers. The unresolved tradeoff between aims and conflicting factors adds complexity to energy optimization problems. Current models frequently focus on a single component, such as energy cost, while disregarding opposing factors like rebound peaks and user satisfaction. Thus, it is crucial to have a complete model that can effectively handle load scheduling challenges by simultaneously handling conflicting objectives. Motivated by this need, the LSC model is developed, employing the OAWDO algorithm to solve energy optimization with flexible load and effectively manage conflicting objectives. The LSC engages users in DR offered by utility providers, providing dual benefits of minimizing consumers electricity bill and mitigating energy demand spikes in the load curve.

The organization of this paper is as follows: Section provides an introduction to the developed system model, Section demonstrates the simulations, and Section presents the conclusion.

## Developed energy optimization model

**Fig 1:**
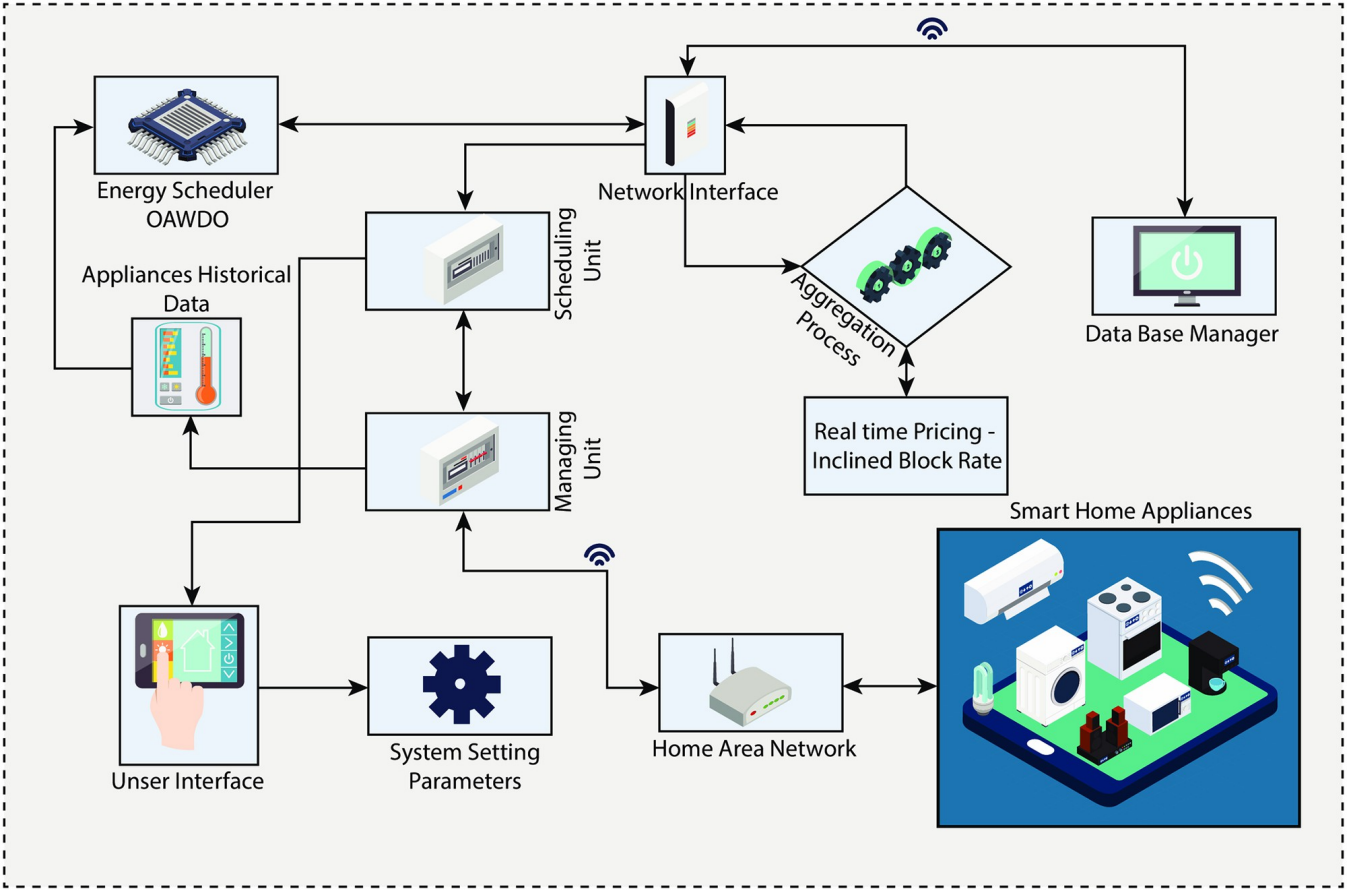
Developed energy optimization model with LSC based on OAWDO algorithm.

The proposed energy optimization model addresses the load scheduling problem in SPG, aiming to optimize electricity bills, mitigate PADR, and alleviate rebound peaks without compromising consumer comfort. The developed framework encompasses components such as advanced metering infrastructure, smart meters, household appliances, SPVE, storage batteries, control and monitoring display, and LSC. The framework is shown in Fig 1. Each component of the developed model is discussed as follows.

The AMI is an essential technology that enables utilities to efficiently monitor and regulate the energy usage of consumers. It also empowers users to participate actively in the electricity market by helping them optimize load scheduling, ultimately improving the reliability of the power grid. This has significant advantages for both users and utility firms. Smart meters (SMs) are advanced electronic devices used for the real-time measurement and logging of energy usage. In contrast to conventional meters, SMs provide bidirectional contact between utility firms and consumers, allowing remote monitoring and control of energy consumption. The household appliances comprising twelve appliances, including a stove, microwave, kettle, vacuum cleaner, electric water heater, toaster, iron, washing machine, tumble dryer, dishwasher, refrigerator, and air conditioner, as sourced from [46]. Appliances like the WM, TD, and dishwasher are shiftable, allowing their operation to be delayed or advanced. The refrigerator and air conditioner are power-shiftable appliances. On the other hand, the electric water heater, iron, and vacuum cleaner are non-shiftable appliances. Lastly, the toaster, stove, microwave, kettle, etc., are considered critical in nature. The specifications and characteristics of household appliances are listed in Table 1 and are sourced from [46]. This study explores solar photovoltaic energy (SPVE) owing to its abundance, easy accessibility, and cost-effectiveness. The SPVE is mathematically modeled and defined in Eq (1), as detailed in [47].

$$\begin{array}{c} P^{pv}(t)=\partial^{pv}\times A^{pv}\times rad(t)\times (1-0.005\times (Tp(t)-25)) \end{array}$$ (1)

**Table 1:** Parameters of smart appliances used in the developed model.

No.	Appliances	Power (kW)	Duration (hrs)	*α*_*i*_	*β*_*i*_	Priority
						04
01	Washing machine	4	3	00:00	04:00	
02	Tumble dryer	3	3.3	05:00	08:00	
03	Dishwasher	2	2.5	22:00	23:59	
						03
04	Air conditions	24	0.8-1.8	00:00 am	23:59	
05	Refrigerator	24	0.5-1.5	00:00 am	23:59	
						02
06	Electric water heater	2	2.6	06:00, 18:00	07:00, 19:00	
07	Vacuum cleaner	3	1.2	10:00	13:00	
08	Iron	2	1.235	08:00	10:00	
						01
09	Toaster	1	1.010	06:00	07:00	
10	Kettle	1	1.9	6:00	07:00	
11	Stove	2	3.0	07:00	09:00	
12	Microwave	1	1.230	13:00	14:00	

The symbols in Eq (1) are defined as $P^{pv}$ represents the power output produced, $\partial^{pv}$ represents the efficiency of the SPVE panel, and $A^{pv}$ shows the area of the solar panel. In addition, *rad*(*t*) represents irradiation and *Tp*(*t*) represents temperature. The temperature compensation factor is a fixed value of 0.005. The probability density function is utilized to represent solar irradiation using Eq (2), as referenced in the publication [47].

$$\begin{array}{c} F(rad(t))=\omega (\frac{\theta 1}{\lambda 1}){{\left(\frac{rad(t)}{\lambda 1}\right)}^{\left(\theta 1-1\right)}}^{e^{-{\left(\frac{rad}{\lambda 1}\right)}^{\theta 1}}}+(1-\omega )(\frac{\theta 2}{\lambda 2}){\left(\frac{rad(t)}{\lambda 2}\right)}^{(\theta 2-1)e^{-{\left(\frac{rad}{\lambda 2}\right)}^{\theta_{2}}}} \end{array}$$ (2)

where the symbols used in Eq (2) have the meanings explained below: The weigh factor is *ω*; the shape factors are *θ*1, *θ*2, indicating that the function *rad*(*t*) is inside the range of positive real integers; and the scale factors are λ1, λ2.

The LSC optimally employs the available SPVE during high-price hours to power loads and stores excess energy in storage batteries during low-price hours. The main goal of the LSC is to optimize the utilization of SPVE, resulting in decreased pollutant emissions and reduced utility expenses.

Storage batteries provide ancillary services to improve the reliability, sustainability, and resilience of the utility grid. In this work, the services utilized by storage batteries include load scheduling, backup power, and integration of renewable energy. For instance, the backup service entails the process of recharging the batteries during periods of reduced demand when electricity usage is lower and discharging stored energy during periods of high demand. Additionally, storage batteries play a role in integrating SPVE into the power grid. Due to the variable and intermittent nature of SPVE, storage batteries play a pivotal role in reducing its fluctuations and balancing the supply and demand of energy. Through the efficient storage of surplus energy generated during peak production and its subsequent release during periods of low production, storage batteries contribute significantly to stabilizing the energy supply system. This capability enhances the reliability of energy provision by mitigating variations and ensuring a consistent flow of electricity to users. Moreover, storage batteries exchange power with utility providers during peak load demand hours, as highlighted in [48]. Hence, storage batteries significantly contribute to achieve objectives such as pollution emissions reduction and notably utility bills alleviation. The storage batteries model is presented in Eq (3), as in [47].

$$\begin{array}{c} E_{SB}(t)=E_{SB}(t-1)+\eta \cdot \mu^{SB}\cdot p_{SB}^{ch}(t)-\frac{\eta \cdot p_{SB}^{dch}(t)}{\mu^{SB}}\forall t \end{array}$$ (3)

Eq (3) illustrates that energy stored in the batteries during a particular period depends on the energy stored in the batteries during the last hours, accounting for the impact of any charging or discharging activities. Eqs (4) and (5) shows that the battery’s charging and discharging power is limited to a specific maximum power range.

$$\begin{array}{c} 0⩽\frac{p_{SB}^{ch}(t)}{\eta_{ch}}⩽p_{ch}^{\text{max}} \end{array}$$ (4)

$$\begin{array}{c} 0⩽p_{SB}^{dch}\left(t\right)\times \eta_{dch}⩽p_{dch}^{\text{max}} \end{array}$$ (5)

The state of charge (SOC) of the battery is managed to extend its overall lifespan. The SOC is presented in Eq (6) and limited between maximum and minimum upper bounds as in (7). When the SOC surpasses the maximum limit, no additional energy is permitted to be stored in the battery. Conversely, if the SOC falls below the minimum threshold, no further energy is drawn from the battery.

$$\begin{array}{c} SOC\left(t+1\right)=SOC\left(t\right)+\frac{p_{SB}^{ch}(t)-p_{SB}^{dch}(t)}{E_{SB}(t)} \end{array}$$ (6)

$$\begin{array}{c} SOC^{\text{min}}⩽SOC\left(t\right)⩽SOC^{\text{max}} \end{array}$$ (7)

where symbol *E*_*SB*_(*t*) represents the energy stored in the battery, while $p_{SB}^{ch}$ and $p_{SB}^{dch}$ denote the charging and discharging power, respectively. Additionally, *η*_*ch*_ and *η*_*dch*_ represent the charging and discharging efficiencies. The state of charge, denoted as *SOC*, is constrained within the upper limit *SOC*^max^ and the lower limit *SOC*^min^, likewise in [49].

This study focuses on DR, which is categorized as price-based and incentive-based DR. In particular, this work delves into the realm of price-based DR, which encompasses various schemes such as RTP, DAP, CPP, ToU, and others, as outlined in [50]. RTP offers greater flexibility than other DRs, making it easier to coordinate the operation of numerous appliances during periods of relatively lower prices. However, in a basic RTP scheme in which all users aim to minimize their electricity bills, mostly home appliances are shifted to hours with lower prices. This results in the creation of rebound peaks, where net load surpasses the IBT, leading to higher costs for residents than anticipated. To address this dilemma, RTP is integrated with IBT and employed in this study, modeled in Eq (8), adopted from [51, 52].

$$\begin{array}{c} \varphi (t)=\{\begin{array}{c} \rho \left(t\right)\;\;\text{if}\;0⩽\varepsilon_{c}^{i}\left(t\right)⩽\varepsilon_{thr}\\ \lambda .\rho \left(t\right)\;\;\text{if}\;\varepsilon_{c}^{i}\left(t\right)⩾\varepsilon_{thr} \end{array} \end{array}$$ (8)

where *ρ*(*t*) is RTP scheme, *φ*(*t*) is combined RTP-IBT scheme, if the $\varepsilon_{c}^{i}\left(t\right)$ is less than the IBT threshold *ε*_*thr*_ the users will be charged as per RTP scheme. On the other hand, if $\varepsilon_{c}^{i}\left(t\right)$ exceeds the IBT threshold *ε*_*thr*_ the users will be charge at higher price than *ρ*(*t*) by constant number λ.

The LSC using OAWDO processes the operation patterns of appliances and generation data from SPVE, storage batteries, and the utility subjected to utility and user constraints, generating a load schedule using RTP-IBT methodology to effectively meet specific goals, such as reducing power bills, minimizing peak demand and rebound peaks, while ensuring consumer satisfaction is not compromised.

### Modelling and formulation

The aim of this study is to tackle the load scheduling issue by optimizing power bills, PADR, and reducing rebound peaks, while ensuring consumer satisfaction is not compromised. Initially, each objective is formulated individually. Consequently, the load scheduling problem is defined as an optimization challenge.

Each appliance, denoted by *i*, is assigned a time span, represented by *T*_*h*_, which ranges from 1 to 24. This time span indicates the scheduling timeframe in which all appliances are controlled, taking into account different limitations. The energy consumption of each appliance is computed using the following formula:

$$\begin{array}{c} \varepsilon_{t}^{i}=p_{t}^{i}\times X_{t}^{i} \end{array}$$ (9)

where symbols $\varepsilon_{t}^{i}$, $p_{t}^{i}$, and $X_{t}^{i}$ show appliance *i* energy consumption at hour *t*, power demand by appliance *i* at hour *t*, and appliance *i* status indicator (ON/OFF), respectively. The net energy used is determined by adding up the energy consumed by each individual appliance.

$$\begin{array}{c} \varepsilon_{T}=\sum_{t=1}^{T}(\sum_{i=1}^{N}p_{t}^{i}\times X_{t}^{i})\times \Delta t \end{array}$$ (10)

In Eq (10), the symbol *ε*_*T*_ represents the total energy consumed throughout the scheduling period. The symbol *Δt* represents the duration of each time interval, and the summation is performed across all intervals in the scheduling period for all appliances. Electricity bills are charges that utility providers impose on consumers for the electricity they use within a certain period of time. The cost of all appliances in terms of power bill is represented by Eqs (11) and (12), which simulate the scenarios without SPVE and batteries, and with SPVE and batteries, respectively.

$$\begin{array}{c} \gamma =\varepsilon_{T}\times \;\varphi \left(t\right)\times \Delta t \end{array}$$ (11)

The net electricity bill is denoted as *γ* without SPVE and batteries, which is calculated using the RTP-IBT signal, which is represented as *φ*(*t*). The calculation of the electricity bill involving SPVE and storage batteries is as follows.

$$\begin{array}{c} \gamma_{SB}^{PV}=(\varepsilon_{T}-\varepsilon_{T}^{PV}-\varepsilon_{T}^{SB})\times \;\varphi \left(t\right)\times \Delta t \end{array}$$ (12)

where the net electricity bill denoted $\gamma_{SB}^{PV}$ with SPVE and batteries.

The PADR is a quantitative measure that indicates the relationship between the highest and average levels of demand within specific time intervals. Reducing the PADR has advantages for both customers and utilities as it helps to minimize the gap between energy supply and demand. The symbol ℜ represents PADR. The PADR can be found in Eq (13), where *ε*_(*t*)_ represents appliances power consumption.

$$\begin{array}{c} ℜ=\frac{{\text{max}}_{t\in T}(\varepsilon_{\left(t\right)})}{\frac{1}{T}\sum_{t=1}^{T}\varepsilon_{\left(t\right)}} \end{array}$$ (13)

This study investigates customer satisfaction with waiting time, primarily focusing on the delay consumers experience when adjusting their appliance usage from peak hours to off-peak hours. User discomfort, measured in units of time, encompasses both delay and advancement of the schedule. The operation pattern of appliances undergoes a shift before and after scheduling, as devices are now operated during low-price timeslots rather than high-price ones. This shift may lead to user frustration, which is quantified in terms of delay. Consumers who are open to waiting a bit longer have the opportunity to save on their electricity bills by adjusting their energy-consuming activities to off-peak hours. Conversely, individuals with limited waiting capacity may face higher electricity costs as they are compelled to utilize energy during peak hours when tariffs are typically higher. This tradeoff between electricity bill optimization and waiting time underscores the importance of consumer preferences and their impact on energy consumption patterns. Appliances with adjustable power settings operate continuously for 24 hours, thereby offering flexibility in power consumption scheduling. This feature enables these appliances to adapt their energy usage according to the demand and availability of electricity, thus contributing significantly to the optimization of scheduling through their power flexibility. and resulting in zero waiting time. The model for consumer comfort related to waiting time is structured in a manner similar to that presented in [50].

$$\begin{array}{c} \kappa =\frac{\sum_{t=1}^{T}\sum_{i=1}^{n}|(T_{i,t}^{bsch}-T_{i,t}^{asch})|}{T_{i}^{l}} \end{array}$$ (14)

The variable *κ* represents the time duration during which each appliance *i* may need to wait before it can modify its operating hours. $T_{i,t}^{bsch}$ and $T_{i,t}^{asch}$ represent the operating condition of appliance *i* before and after scheduling, respectively. The variable $T_{i}^{l}$ represents the overall duration of appliance operation. The LSC coordinates the adjustment and shifting of appliances from high-price to low-price hours, taking into account RTP-IBT and consumer preferences. The main objective of load scheduling is to evenly distribute electricity in order to address the difference between demand and generation. The goal is to minimize electricity costs and power outages while ensuring that consumer satisfaction is maintained. In order to address these goals, the load scheduling problem is approached as an optimization challenge. The objective function is a combination of weighted factors including electricity bills, PADR, and consumer comfort.

$$\begin{array}{c} Min(f)=w_{1}\gamma_{SB}^{PV}+w_{2}ℜ+w_{3}\kappa \end{array}$$ (15)

where *f* is the objective function, *w*_1_, *w*_2_, and *w*_3_ are non-negative weights corresponds to electricity bill, PADR, and user discomfort. The load scheduling problem, as defined by Eq (15), is governed by the following set of constraints.

$$\begin{array}{c} p_{t}^{g}+p_{t}^{PV}=p_{t}^{l}+p_{SB}^{ch}(t)-p_{SB}^{dch}(t) \end{array}$$ (16a)

$$\begin{array}{c} E_{SB}^{\text{min}}⩽E_{SB}\left(t\right)⩽E_{SB}^{\text{max}} \end{array}$$ (16b)

$$\begin{array}{c} \varepsilon_{T}^{bsch}=\varepsilon_{T}^{asch} \end{array}$$ (16c)

$$\begin{array}{c} T_{i}^{l,bsch}=T_{i}^{l,asch} \end{array}$$ (16d)

$$\begin{array}{c} X_{t}^{i,bsch}\neq T_{t}^{i,asch} \end{array}$$ (16e)

Constraint (16a) indicates that left-hand side incorporates positively signed terms for the power obtained from the grid and the SPVE. On the right-hand side, the power consumed by loads (appliances), as well as the charging power of the storage batteries, is depicted with a positive sign, whereas the discharging power is indicated by a negative sign. Constraint (16b) ensures that the energy stored in the battery must be bounded between the upper and lower bounds. Constraint (16c) ensures that the energy consumption is constant before and after scheduling. Constraint (16d) ensures that duration length of operation must be equal before and after scheduling. Similarly, constraint (16e) ensures that appliances operation status before and after scheduling must not be equal.

### Proposed OAWDO technique

This section provides an overview of the developed OAWDO algorithm. The OAWDO algorithm is inspired by the work presented in [53]. It is employed within the LSC to address energy optimization challenges involving flexible load management and DR. The primary aim is to achieve the desired objectives of minimizing electricity expenses and PADR while ensuring the user satisfaction is maintained. The parameters of the OAWDO algorithm are as follows: *N* denotes the number of air parcels, while *D* represents the count of decision variables targeted for optimization. Velocity bounds are set by *v*_max_ and *v*_min_, determining the maximum and minimum velocities for the air parcels, respectively. Similarly, position limits are defined by *x*_max_ and *x*_min_, specifying the maximum and minimum positions. The number of runs, denoted by *n*_runs_, indicates how many iterations or runs the algorithm undergoes, with each run comprising *n*_itr_ iterations. Parameters such as *RT*, *α*, *g*, and *c* are pertinent to the optimization process. Additionally, *p*_*c*_ and *p*_*m*_ represent the crossover and mutation probabilities, respectively. The optimization algorithm utilizes an initial weight *w*_*i*_, which gradually transitions to *w*_*f*_ as the algorithm progresses. Adjusting these parameters can significantly influence the algorithm’s convergence speed, exploration-exploitation balance, and overall effectiveness in optimizing the problem at hand. A nature-inspired optimization algorithm known as the WDO has been created to emulate wind dynamics within the atmosphere. The WDO encompasses forceful and non-aggressive regions, employing velocities that correlate with pressure gradient forces. Virtual air parcels are expressed through a position vector presented in Eq (17), where *N* denotes air parcels count and *D* shows decision variables count targeted for optimization, as follows.

$$X=\left[\begin{array}{llll} x_{11} & x_{12} & \cdots & x_{1D}\\ x_{21} & x_{22} & \cdots & x_{2D}\\ \vdots & \vdots & \ddots & \cdots \\ x_{N1} & x_{N2} & \cdots & x_{ND} \end{array}\right]$$ (17)

Subsequently, air parcels fitness values are presented as fitness vector in Eq (18) given below.

$$\begin{array}{c} f\left(x\right)=[\begin{array}{c} f([x_{1,1}\;x_{1,2}\;\cdots \;\;\;x_{1,D}])\\ f([x_{2,1}\;x_{2,2}\;\cdots \;\;x_{2,D}])\\ \;\vdots \;\;\;\;\;\vdots \;\;\;\;\ddots \;\;\cdots \\ f([x_{N,1}\;x_{N,2}\;\cdots \;\;x_{N,D}]) \end{array}] \end{array}$$ (18)

In Eq (18), the entry *f*(…) row signifies the fitness value associated with *x* air parcel. The WDO adheres to Newton’s 2nd law of motion, asserting that the acceleration of air parcel is contingent on *F*_*t*_, which is the overall force and reciprocally on *ρ*, which is its air density. As the magnitude of the *F*_*t*_ increases, *ω* increases, which is the parcel acceleration. Conversely, when the air density expands, the acceleration experiences a reduction, expressed as

$$\begin{array}{c} \rho \omega =\sum F_{t}={\text{F}}_{\text{G}}+{\text{F}}_{\text{PG}}\;\text{+}\;{\text{F}}_{\text{C}}+{\text{F}}_{\text{F}} \end{array}$$ (19)

The motion of air parcels results from the total force ∑*F*_*t*_ including gravitational force (F_G_ = *ρδv*_*g*_), pressure gradient force (F_PG_ = −Δ*p* × *δV*), Coriolis force (F_C_ = −2Ω × *v*), and frictional force (F_F_ = −*ρα*_*f*_ × *v*). Here, *g* denotes gravitational acceleration, *δV* represents a finite volume of air parcel, Δ*p* represents the pressure gradient, Ω signifies Earth’s rotation, *v* is wind velocity vector, and *α*_*f*_ denotes friction coefficient. Now, substitute F_G_, F_PG_, F_C_, F_F_ into Eq (19), and the resulting equation will be as follows.

$$\begin{array}{c} \rho \omega =\rho \delta vg+(-\Delta p\times \delta V)+(-2\Omega \times v)+(-\rho \alpha_{f}\times v) \end{array}$$ (20)

Assuming, for simplicity, that air parcel acceleration is expressed as $\omega =\frac{\Delta V}{\Delta t}$, with Δ*t* = 1 and the change in velocity Δ*V* set to 1 for a small air parcel, substituting these values into Eq (20) yields the following revised equation.

$$\begin{array}{c} \rho \Delta v=(\rho \times g)+(-\Delta p)+(-2\Omega \times v)+(-\rho \alpha_{f}\times v) \end{array}$$ (21)

Relationship between air pressure denoted by *p*, density represented by *ρ*, and temperature *T* is established by the ideal gas law, expressed as *p* = *ρRT*. Substituting $\rho =\frac{p}{RT}$ into the equation and relocating it to the right-hand side results in:

$$\begin{array}{c} \Delta v=g+(-\Delta p\frac{RT}{p_{t+1}})+(-2\Omega \times v_{t}^{i}\times \frac{RT}{p_{t+1}})+(-\alpha_{f}\times v_{t}^{i}) \end{array}$$ (22)

where $\Delta v=V_{t+1}^{i}-V_{t}^{i}$ represents the change in velocity between the previous $V_{t}^{i}$ and current states $V_{t+1}^{i}$, *R* denotes the universal gas constant, and *p*_*t*+1_ denotes the air parcel’s pressure at its current position, replacing the variable *p*. The WDO algorithm enhances the position and velocity of air parcels guiding them towards the optimal pressure position during iteration. Ultimately, as the iterations progress, WDO converges to the optimal solution. The Eq (22) for update velocity is modified as follows.

$$\begin{array}{c} V_{t+1}^{i}=(\left(1-\alpha_{f}\right)\times V_{t}^{i})-g+(-\Delta p\frac{RT}{p_{t+1}})+(-2\Omega \times v_{t}^{i}\times \frac{RT}{p_{t+1}}) \end{array}$$ (23)

Let *g* and *δp* be vectors that can be decomposed into both direction and magnitude as $g=|g|(0-X_{t+1}^{i}),\;\Delta p=-|p^{best}-p_{t+1}|\left(X_{t}^{best}-X_{t}^{i}\right)$. The *p*^*best*^ denotes the optimal pressure point discovered, pressure at the present position is *p*_*t*+1_, $X_{t+1}^{i}$ represents the current position of air parcel in the ongoing iteration, and $X_{t}^{best}$ signifies previous global optimal position. Moreover, the effect of the Coriolis force, *ω* × *v*, is overshadowed by the indiscriminate effect of velocity from a different dimension, represented as $V_{t}^{i,other}$, which enhances the movement of the parcel. Subsequently, *c* = −2*RT*, and air parcels are arranged based on pressure values, with *i* representing ranks within the population. Lower- and upper-pressure values correspond to favorable solution and unfavorable solutions, respectively. In cases where the pressure becomes excessive due to notably increased velocities, thereby diminishing the WDO efficacy, the current pressure is substituted by rank *i* of all air parcels relative to pressure values, then Eq (23) enhances air parcels velocity as follows.

$$\begin{array}{c} V_{t+1}^{i}=(\left(1-\alpha_{f}\right)\times V_{t}^{i})-g\times X_{t}^{i}+(RT|1-\frac{1}{i}|\left(X_{t}^{best}-X_{t}^{i}\right))+(\frac{c\times V_{t}^{i,other}}{i}) \end{array}$$ (24)

The position of an air parcel is formulated as follows.

$$\begin{array}{c} X_{t+1}^{i}=X_{t}^{i}+(V_{t+1}\times \Delta t) \end{array}$$ (25)

The position and velocity air parcels undergo enhancement during iterations, utilizing the respective equations based on existing pressure values. Following this, the parcels transition from high-pressure to low-pressure regions, akin to the movement of wind in the atmosphere. The conclusion of iterations in the WDO algorithm can occur upon reaching a predefined target for the pressure values or upon reaching the maximum specified number of iterations.

A novel optimal adaptive wind-driven optimization (OAWDO) algorithm is developed by incorporating two enhancements from enhanced differential evolution algorithm into the wind-driven optimization (WDO) algorithm. Firstly, a crossover is employed to improve WDO exploration ability. Secondly, the mutation operator is integrated to improve the exploitation ability of WDO. This improvement results in the developed algorithm, namely OAWDO, which explores the most promising regions it identifies and possesses the ability to avoid local optima. Utilizing the developed OAWDO, an updated population is created by selecting the most exceptional pairs from the available generation. The superior solutions are preserved based on their evaluation through a fitness function, serving as our objective function. During each iteration of the OAWDO, the air parcels undergo sorting in ascending order according to their fitness values, with the parcel exhibiting the top performance designated named optimal. The crossover operator of OAWDO is applied to population returned from WDO, which is global optimal population of air parcels. This process generates 2 offspring from parents chosen in a manner that involves exchanging segments of the parent strings. The resulting offspring is then added to the population, contributing to the pool of solutions Passed on to the subsequent generation. Ultimately, the global best parcels are selected from solution space, forming updating population for subsequent epoch. It is worth noting that this method helps to avoid retaining the worst-performing air parcels, gradually improving the overall quality of individuals solution space to return global optimal population. Moreover, the OAWDO, with its exploratory characteristics, introduces a delay in its tendency to prematurely converge to local optima. The mutation operator of genetic algorithm integrated with WDO which introduces randomness into the population, enabling occasional exploratory moves in the search space. This helps prevent the algorithm from getting stuck in local optima and contributes to the exploitation of the most promising regions of the solution space. The OAWDO is presented in algorithm 1 and 1.

**Algorithm 1** Part1: OAWDO-based load scheduling for energy optimization using DR

**Data**: Number of air parcels *N*, Number of decision variables *D*, Universal gas constant *R*, Gravitational acceleration *g*, Friction coefficient *α*_*f*_, Earth’s rotation rate Ω, Maximum iterations *MaxIter*

**Result**: Optimal load schedule

Initialize population *X* with *N* air parcels and *D* decision variables Initialize velocities *V* for each air parcel Set parameters *R*, *g*, *α*_*f*_, Ω, *MaxIter* Initialize best pressure *p*_*best*_ and best position *X*_*best*_ Evaluate fitness *f*(*X*) for all air parcels

**for** *t* ← 1 ***to*** *MaxIter* **do**

 **for** *i* ← 1 ***to*** *N* **do**

  Compute forces:

$$F_{G}=\rho \cdot \delta \cdot g$$

$$F_{PG}=-\Delta p\cdot \delta V$$

$$F_{C}=-2\Omega \cdot v$$

$$F_{F}=-\rho \cdot \alpha_{f}\cdot v$$

  Total force:

$$F_{t}=F_{G}+F_{PG}+F_{C}+F_{F}$$

  Update velocity:

$$V_{t+1}^{i}=\left(1-\alpha_{f}\right)\cdot V_{t}^{i}-g\cdot X_{t}^{i}+RT|1-\frac{1}{i}|\cdot \left(X_{t}^{best}-X_{t}^{i}\right)+\frac{c\cdot V_{t}^{i,other}}{i}$$

  Update position:

$$X_{t+1}^{i}=X_{t}^{i}+V_{t+1}^{i}\cdot \Delta t$$

$X_{t+1}^{i}\leftarrow \text{ensure\_constraints}\left(X_{t+1}^{i}\right)$

 **end**

 Evaluate fitness:

f(X)=evaluate_fitness(X)

 Sort air parcels by fitness *X* ← sort_by_fitness(*f*(*X*)) *V* ← *V*[sorted_indices]

 *X*_*new*_ ← [] **for** *j* ← 1 ***to*** *N* ***by*** 2 **do**

  *parent*1 ← *X*[*j*] *parent*2 ← *X*[*j* + 1] *offspring*1, *offspring*2 ← crossover(*parent*1, *parent*2) *offspring*1 ← mutate(*offspring*1) *offspring*2 ← mutate(*offspring*2) *X*_*new*_.append([*offspring*1, *offspring*2])

 **end**

 *X* ← *X*_*new*_

 Update global best: **if** $f_{X}\left[0\right]<f_{X}^{best}$ **then**

  *X*_*best*_ ← *X*[0] *p*_*best*_ ← evaluate_pressure(*X*_*best*_)

 **end**

 **if** *has_converged*(*X*) **then**

  **break**

 **end**

**end**

**return** *X*_*best*_

**Algorithm 1** Part2: OAWDO-based load scheduling for energy optimization using DR

**Function** evaluate_fitness(*X*):

 fitness ← [] **foreach** *x* ∈ *X* **do**

$\gamma_{SB}^{PV}\leftarrow \text{calculate\_gamma\_SB\_PV}\left(x\right)$ ℜ ← calculate_Re(*x*) *κ* ← calculate_kappa(*x*) $f_{x}\leftarrow w_{1}\cdot \gamma_{SB}^{PV}+w_{2}\cdot ℜ+w_{3}\cdot \kappa$ fitness.append(*f*_*x*_)

 **end**

 **return** *fitness*

**Function** ensure_constraints(*X*_*i*_):

 **return** *X*_*i*_

**Function** crossover (*parent*1, *parent*2):

 crossover_point ← random.randint(1, *D* − 1) *offspring*1 ← *parent*1[:

 *crossover*_*point*] + *parent*2[*crossover*_*point*:] *offspring*2 ← *parent*2[:

 *crossover*_*point*] + *parent*1[*crossover*_*point*:] **return** *offspring*1, *offspring*2

**Function** mutate (*offspring*):

 mutation_point ← random.randint(0, *D* − 1) *offspring*[*mutation*_*point*] ← random_value() **return** *offspring*

**Function** has_converged(*X*):

 **return** *False*

**Function** evaluate_pressure(*X*):

 **return** *pressure*

## Simulations, obtained results, and discussions

**Fig 2:**
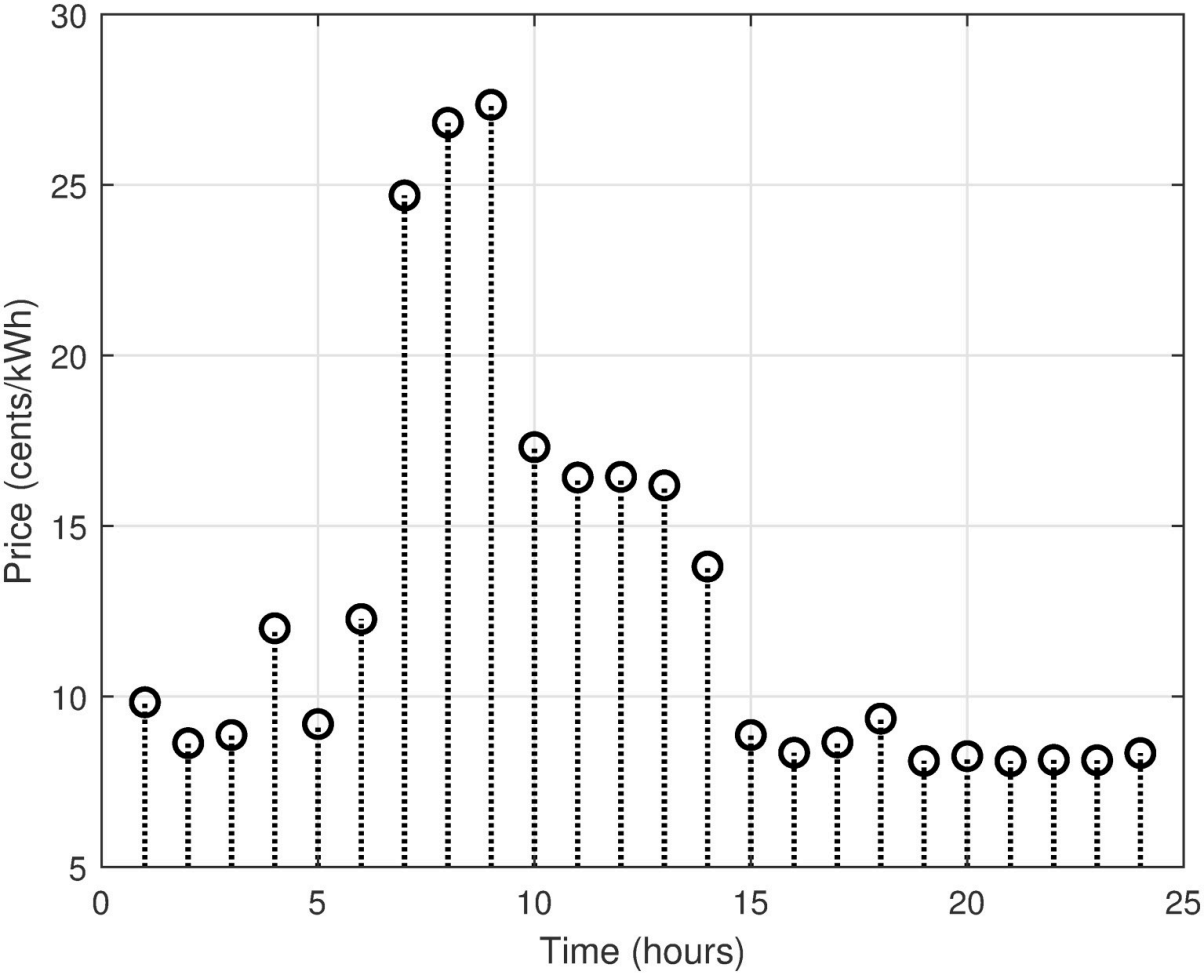
Utility pricing scheme.

**Fig 3:**
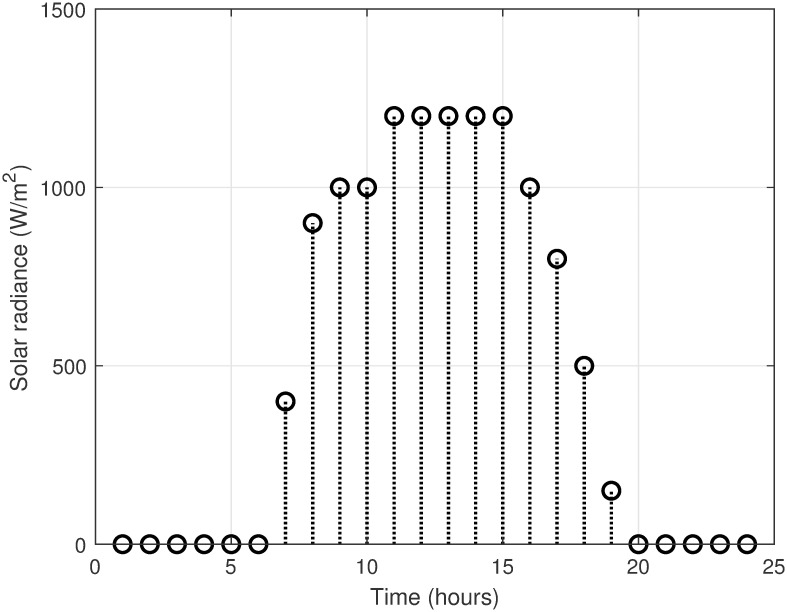
SPVE hourly varying irradiance.

**Fig 4:**
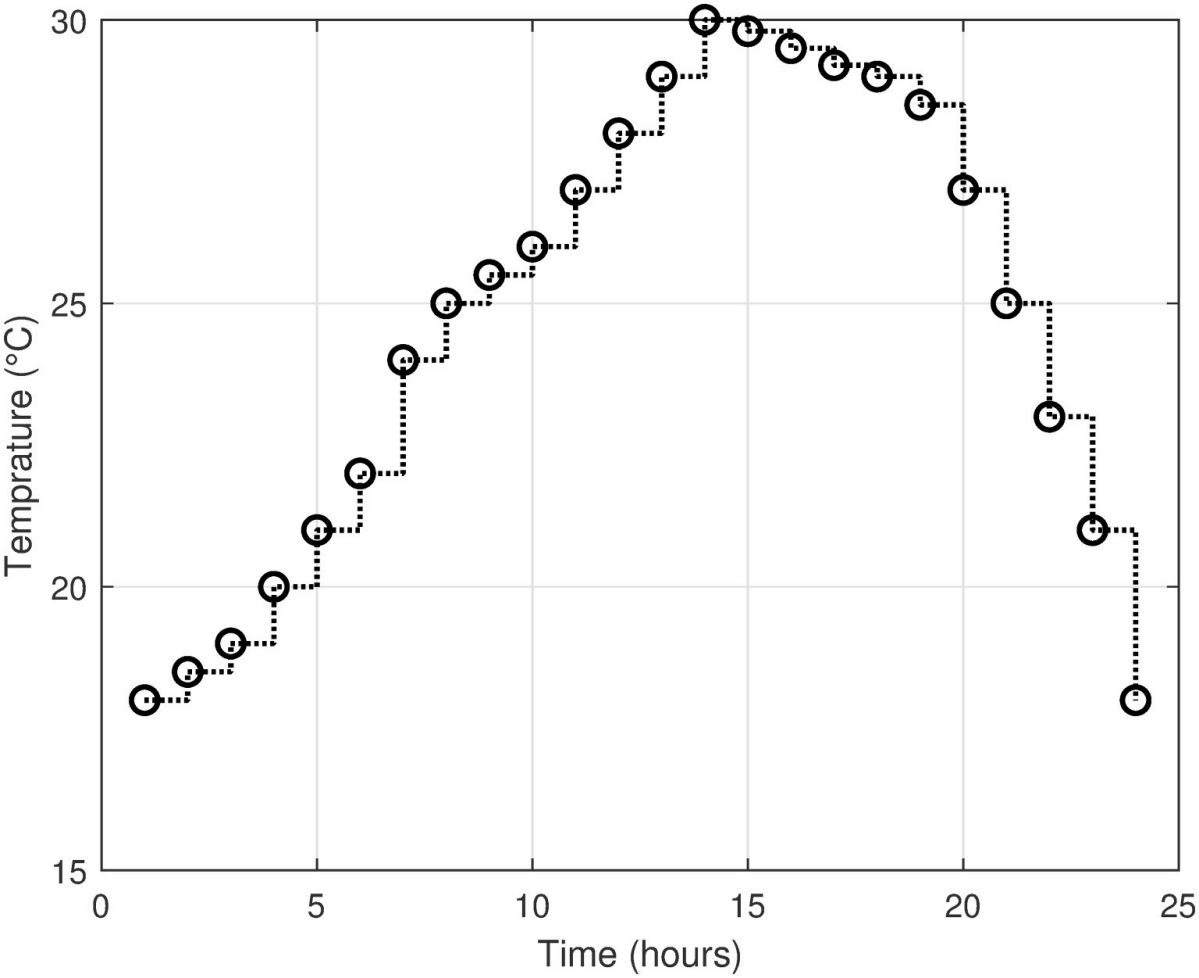
Hourly varying ambient temperature.

**Fig 5:**
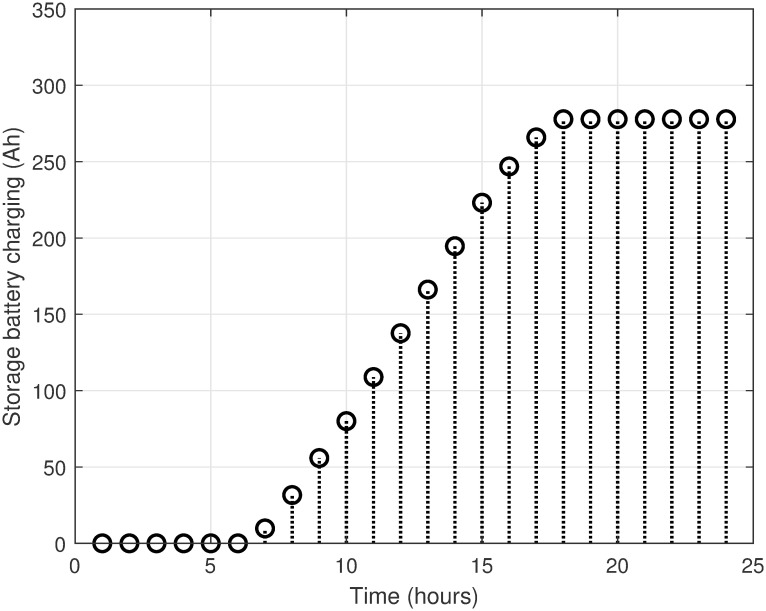
Storage batteries charging level.

**Fig 6:**
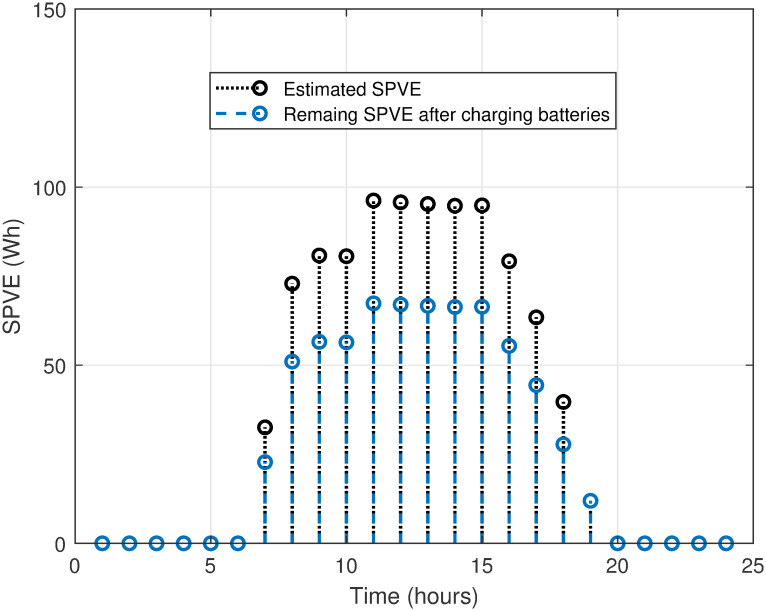
Estimated SPVE generation.

The MATLAB implementation of the created framework, which includes the LSC based on the OAWDO, is compared to several other algorithms such as GA, WOA, FFOA, and WDO. The purpose of this comparison analysis is to verify the efficacy and suitability of the established framework. The system utilizes SPVE and storage batteries to address the load scheduling problem and achieve objectives such as lowering electricity expenditures and PADR while assuring consumer comfort. These components enhance the power supply to the utility system when electricity rates are high, thus optimizing energy use. The OAWDO-based system is implemented with SPVE and storage batteries for performance evaluation in two cases: without scheduling; and with scheduling. The framework includes utility grid, SPVE, and storage batteries, in addition to RTP-IBT and household appliances. These components work together to provide a load-scheduling pattern that maximizes energy efficiency for users. The price signal RTP-IBT of the utility, obtained from the source [51], is depicted in Fig 2. The developed algorithm employs this signal to create a load schedule for the home appliances. Considering the SPVE as a renewable source dependent on solar radiation and temperature, which are depicted in Figs 3 and 4, respectively. Fig 5 displays the charging level of the storage batteries, while Fig 6 showcases the estimated SPVE generation.

The developed LSC, using the OAWDO algorithm, proactively involves users in RTP-IBT to generate a load schedule that considers both SPVE and utility generation. This engagement occurs in two scenarios: one involving the integration of SPVE and storage batteries without scheduling and the other incorporating scheduling for both SPVE and storage batteries. The outcomes for each scenario are elaborated in the subsequent discussion.

### Case I: SPVE and storage batteries integration without scheduling

**Fig 7:**
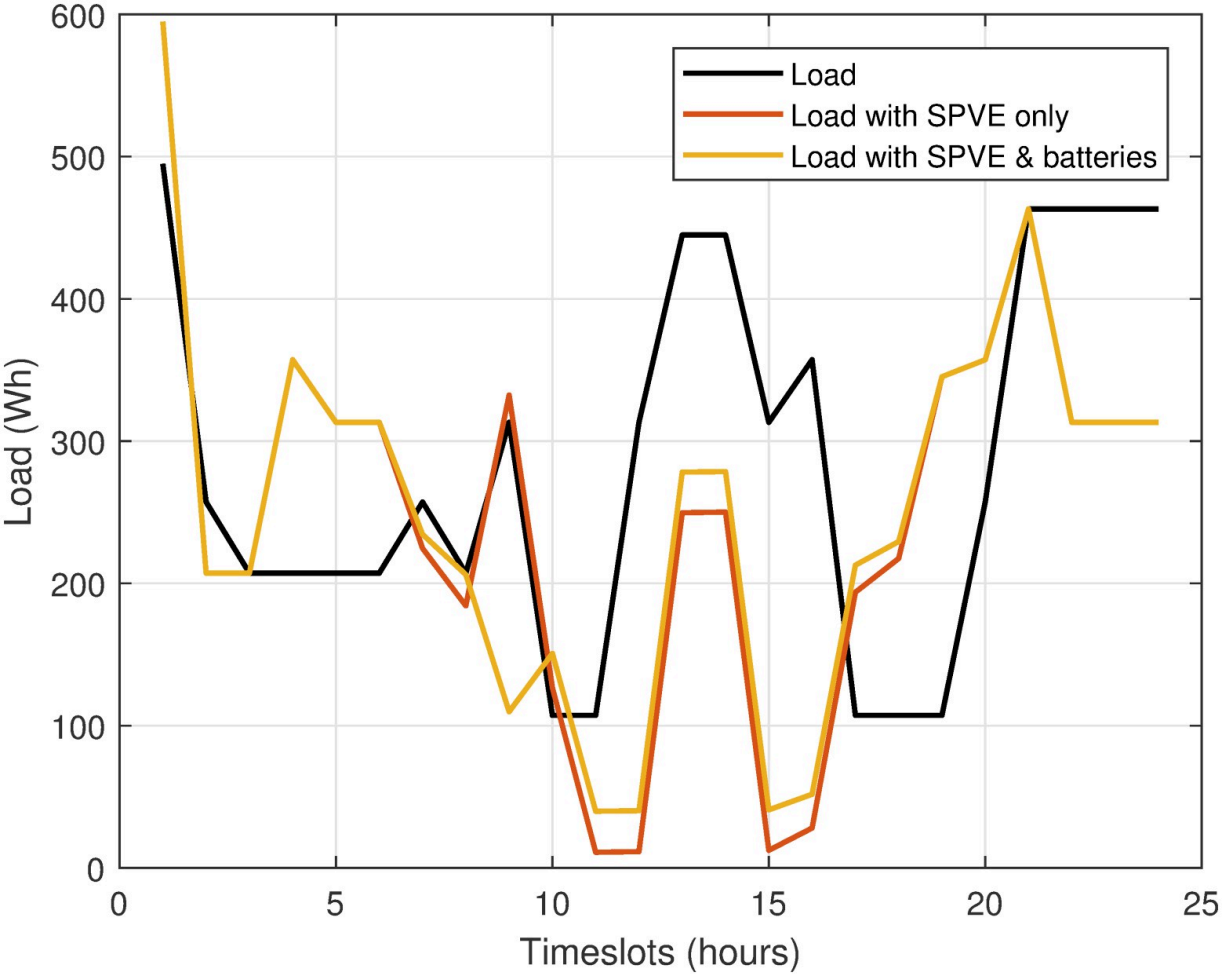
Case 1: Hourly load distribution among utility, SPVE, and SPVE with storage batteries without scheduling.

Case I explores the integration potentials of SPVE and storage batteries without incorporating scheduling considerations related to electricity bills and PADR. The distribution of the load among utility, SPVE, SPVE and storage batteries, and the utility is visualized in Fig 7. This figure highlights the effective shift of a portion of the load from the power grid to SPVE, with SPVE and storage batteries collectively catering to the load during peak hours. The patterns in Fig 7 depict hourly varying loads without scheduling for utility, SPVE, and SPVE with storage batteries. The utility serves a high load during hours 1, 12-14, and 21-24 at 495, 435, and 470W, respectively, and a moderate load during hours 9 and 16 at 305 and 330W. Conversely, a low load of 103W is observed during hours 11 and 16-19. SPVE serves the highest load during hours 9 and 14, while the lowest load is observed at hours 11 and 16, with moderate loads during the remaining hours. The integration of SPVE and storage batteries serves the highest load of 590, 370, and 470 W at periods 1, 4, and 22, respectively. Conversely, it serves the minimum load at hours 11 and 16, with moderate loads during the remaining hours. Notably, the introduction of storage batteries results in a higher overall load serving compared to SPVE and utility.

**Fig 8:**
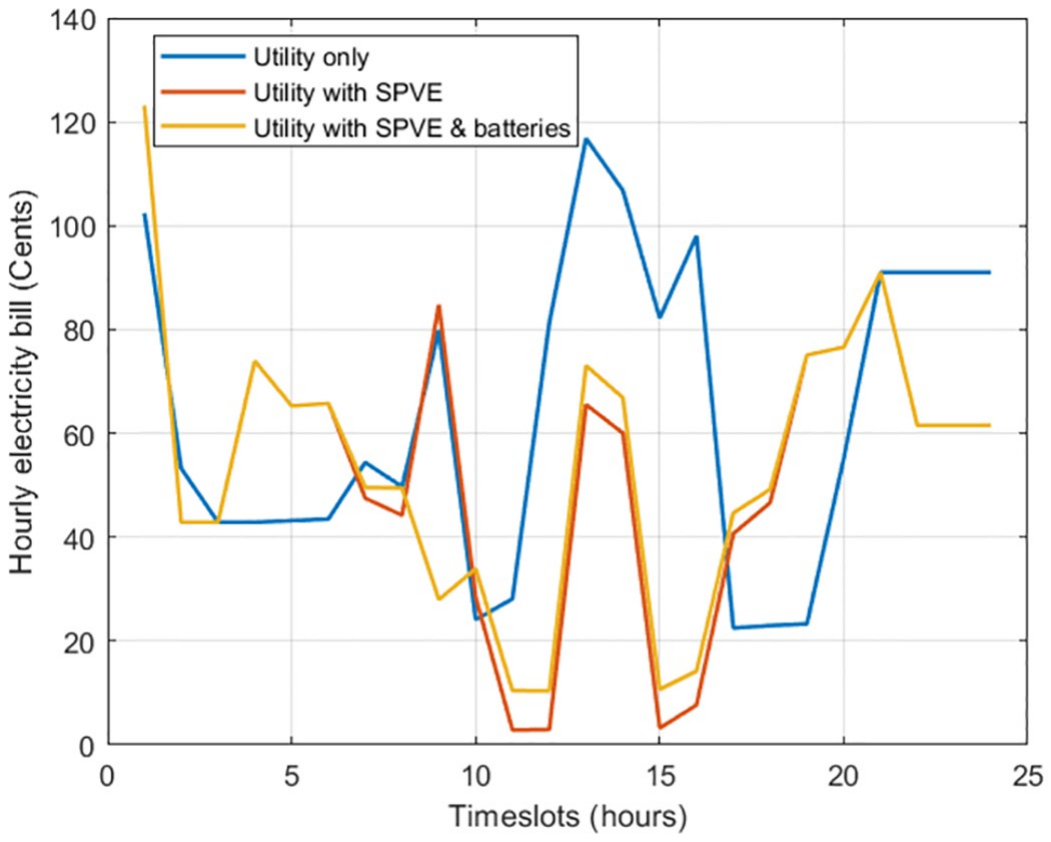
Case 1: Hourly electricity bill minimization without scheduling.

Fig 8 illustrates the hourly electricity bill corresponding to consumed energy without scheduling. Analysis of the data revealed the following key findings. In the case without the SPVE and storage battery integration, during hours 13-14, when the SPVE and storage batteries are not integrated, electricity bills is notably high, amounting to 118 cents.

In a scenario where SPVE integration is implemented, the hourly electricity bill is reduced compared to the scenario where SPVE and storage battery integration are absent. The most significant reduction, from 118 to 65 cents, is observed during hours 13-14 when the SPVE is actively generating power, resulting in a decreased reliance on grid electricity.

In the case of SPVE with storage battery integration, integrating both the SPVE and storage batteries reduces the electricity bill compared to utility only. However, this reduction is lower than the case with only SPVE integration. The combined effect of utilizing an SPVE during daylight hours and leveraging stored energy from batteries during peak periods contributes significantly to the overall reduction in electricity costs.

The findings underscore that the integration of the SPVE and storage batteries is highly effective in reducing the hourly electricity bill. This is particularly evident during peak demand hours, emphasizing the economic benefits of adopting SPVE and energy storage solutions. The analysis supports the notion that a well-designed integration strategy can lead to substantial bill savings and increased sustainability of electricity consumption.

**Fig 9:**
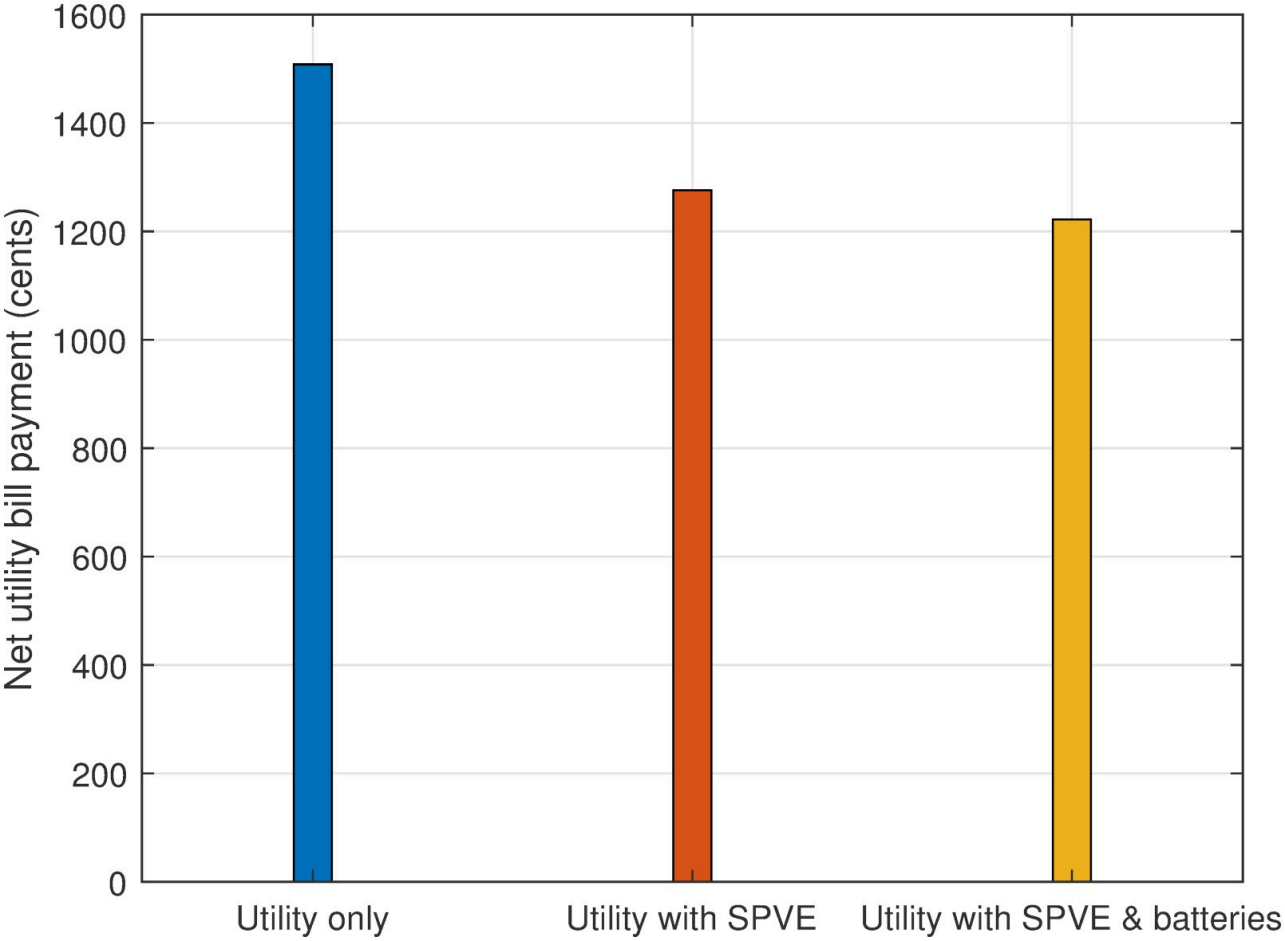
Case 1: Total electricity bill without scheduling.

The total electricity bill minimization without scheduling for case 1 is depicted in Fig 9. Results indicate that incorporating SPVE leads to a 15.17% reduction in the electricity bill. Furthermore, the integration of storage batteries amplifies the cost savings, resulting in a 16.89% overall reduction in the bill. This substantial decrease in electricity expenses can be achieved by making a modest capital investment in the integration of SPVE and storage batteries.

Therefore, the incorporation of SPVE and storage batteries into the system will lead to a per-hour and net reduction in the electricity bill compared to a scenario without the integration of SPVE and storage batteries.

**Fig 10:**
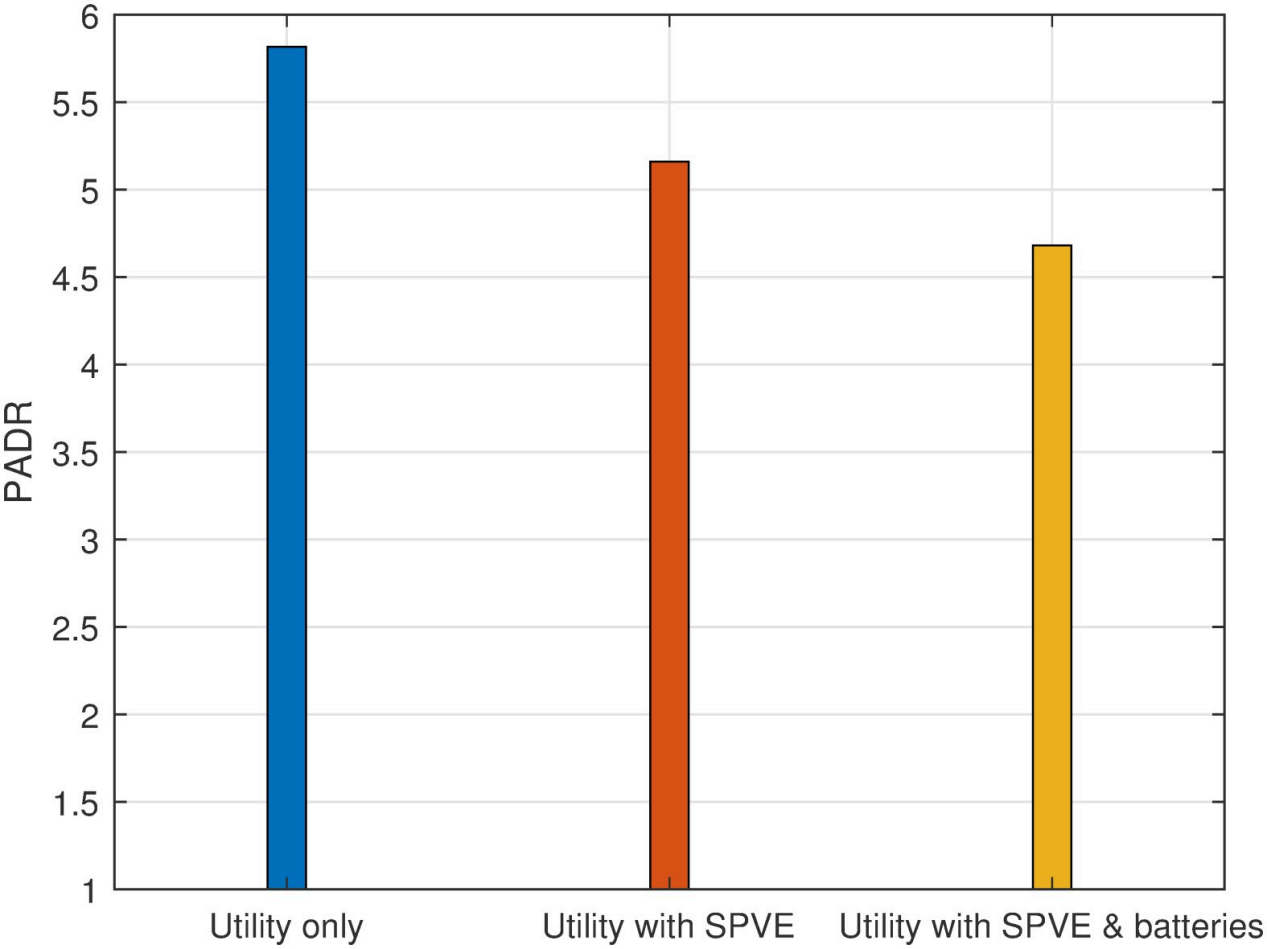
Case 1: PADR evaluation without scheduling.

The evaluation of PADR is presented in Fig 10, considering scenarios with no scheduling and with or without the integration of SPVE and storage batteries. The results indicate that the incorporation of SPVE reduced PADR by 13.79%. Furthermore, the integration of both SPVE and storage batteries into the system results in a more significant reduction in PADR, amounting to 20.68%. Consequently, the integration of SPVE and storage batteries contributes to a decrease in PADR, offering benefits to utility companies in terms of power system reliability improvement and to consumers by lowering electricity bills.

### Case 2: SPVE and storage batteries with scheduling

**Fig 11:**
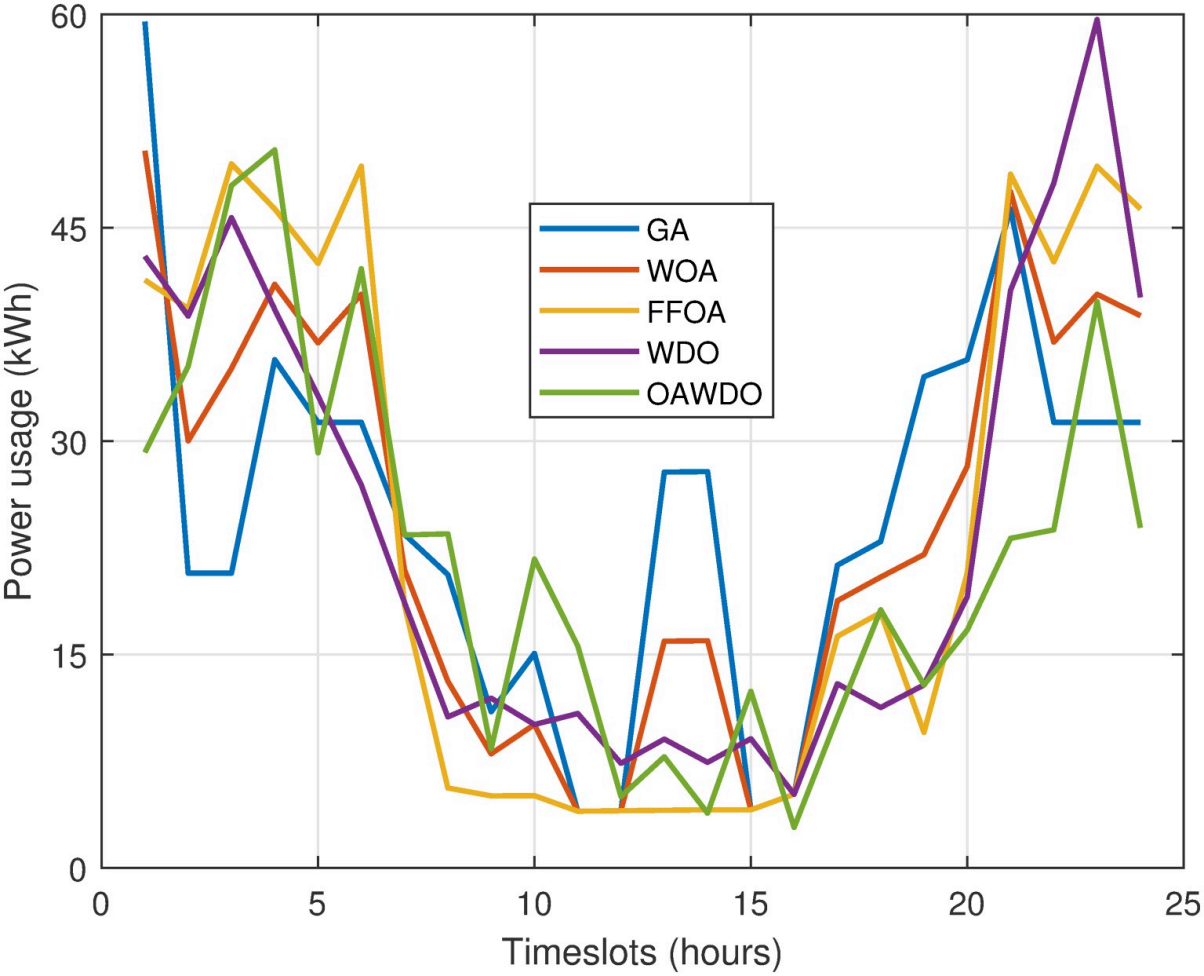
Case 2: Hourly scheduled load profile.

In case 2, the constructed model using the OAWDO is evaluated against alternative methods for load scheduling that incorporate SPVE and storage batteries. The results are discussed below: The load patterns, both scheduled and unscheduled, with and without the integration of SPVE and storage batteries, are illustrated in Fig 11. The GA-based scheduled load pattern with SPVE and storage battery integration exhibited its highest peak at 59 kW during hour 1, and subsequently, there was a maximum power demand of 46 kilowatts at the 20th hour. Medium load patterns of GA-based schedules are observed during time slots 4–6, 12–14, and 21-24, with the lowest load pattern occurring during the remaining hours. Specifically, the maximum load for WOA is 49 kW at hour 1, for FFOA, it is 48.12 kW at hours 4, 6, 21, and 23, for WDO, it is 59.02 kWh at hours 21-22, and for OAWDO, it is 47.14 kW at hours 3-4. The findings depicted in Fig 11 unequivocally demonstrate that the load pattern produced by the developed OAWDO algorithm is both mild and well-balanced in comparison to other existing methods. The OAWDO algorithm efficiently and evenly redistributes the workload from the high-demand hours of 6-14 to the low-demand hours of 1-8 and 19-24 within the scheduling time frame. In contrast to other algorithms, which either shift the workload solely to periods of low demand or manage high workloads during periods of high demand, OAWDO ensures a uniform distribution of the workload. As a result, the OAWDO algorithm generates load patterns that are optimal, making a major contribution to lowering power bills and PADR.

**Fig 12:**
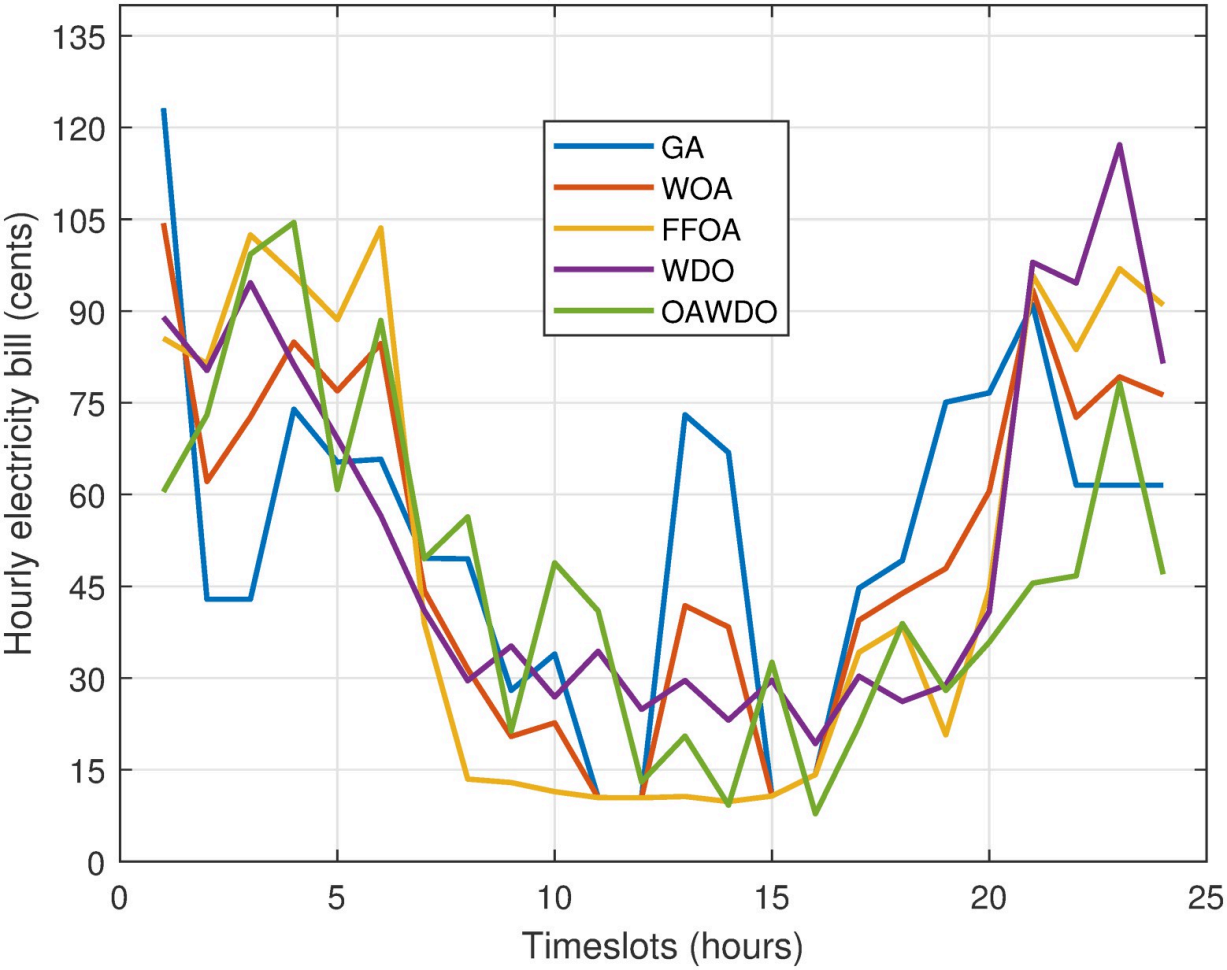
Case 2: Hourly electricity bill with load scheduling.

**Fig 13:**
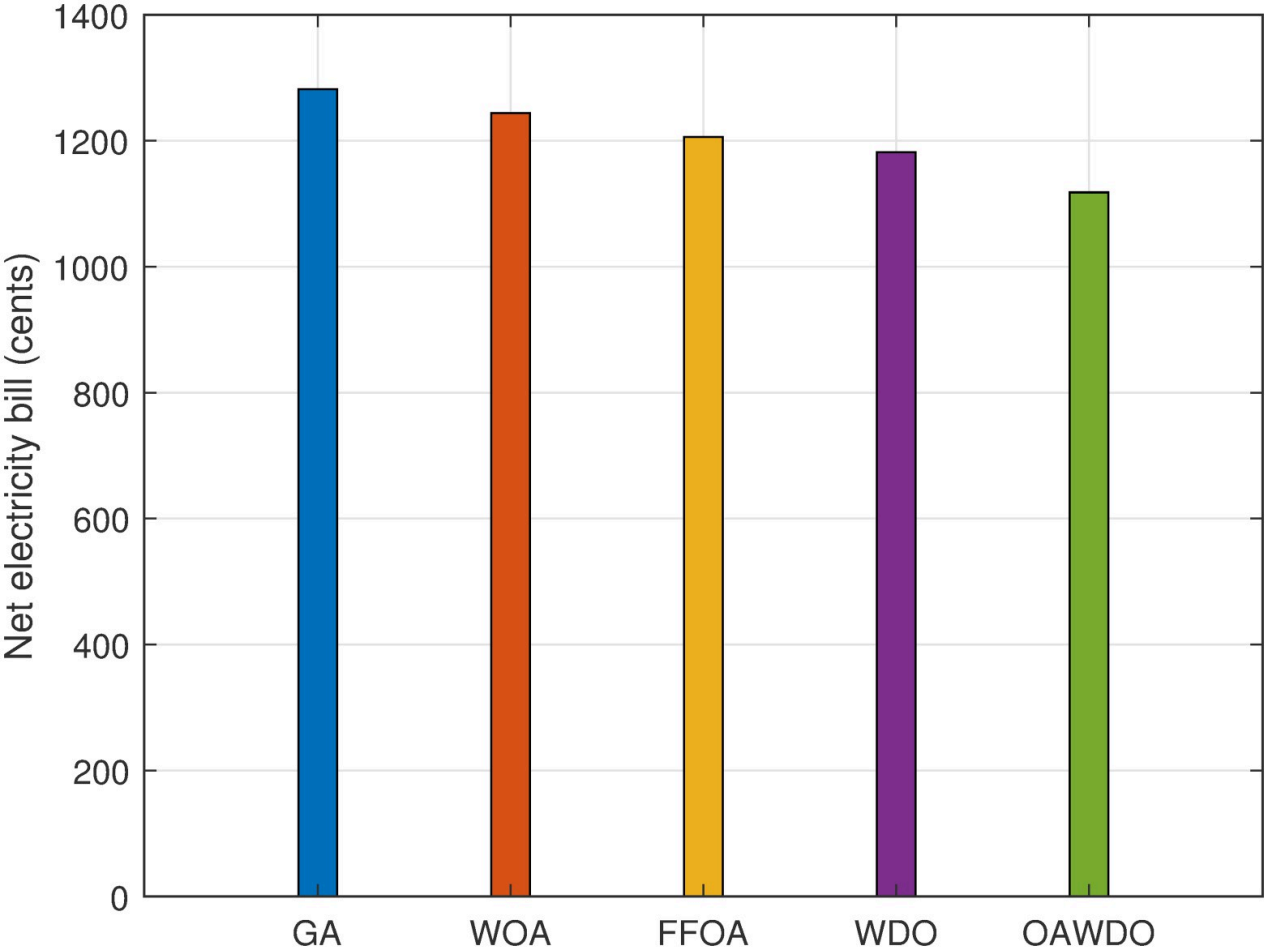
Case 2: Total electricity bill with load scheduling.

Fig 12 illustrates the hourly electricity bill for the energy utilized after scheduling the load using SPVE and storage batteries. The peak electricity bills are as follows: GA at 121 cents during hour 1, WOA at 104.99 cents during hour 1, FFOA at 103.89 cents during hours 3 and 6, WDO at 119.02 cents during hours 21-22, and OAWDO at 104.6 cents during hours 3-4. The profile of the OAWDO algorithm is stable compared to existing algorithms. The total electricity bill for 24 hours of scheduled load using GA, WOA, FFOA, WDO, and OAWDO is shown in Fig 13. The net electricity bill for scheduled load using OAWDO is 1095 cents, while for GA, WOA, FFOA, and WDO algorithms, it is 1330, 1220, 1200, and 1180 cents. The findings indicate that the OAWDO generates a lower electricity expense compared to competing algorithms. The obtained findings confirm that the devised OAWDO algorithm is highly effective in reducing both hourly and net electricity expenses.

**Fig 14:**
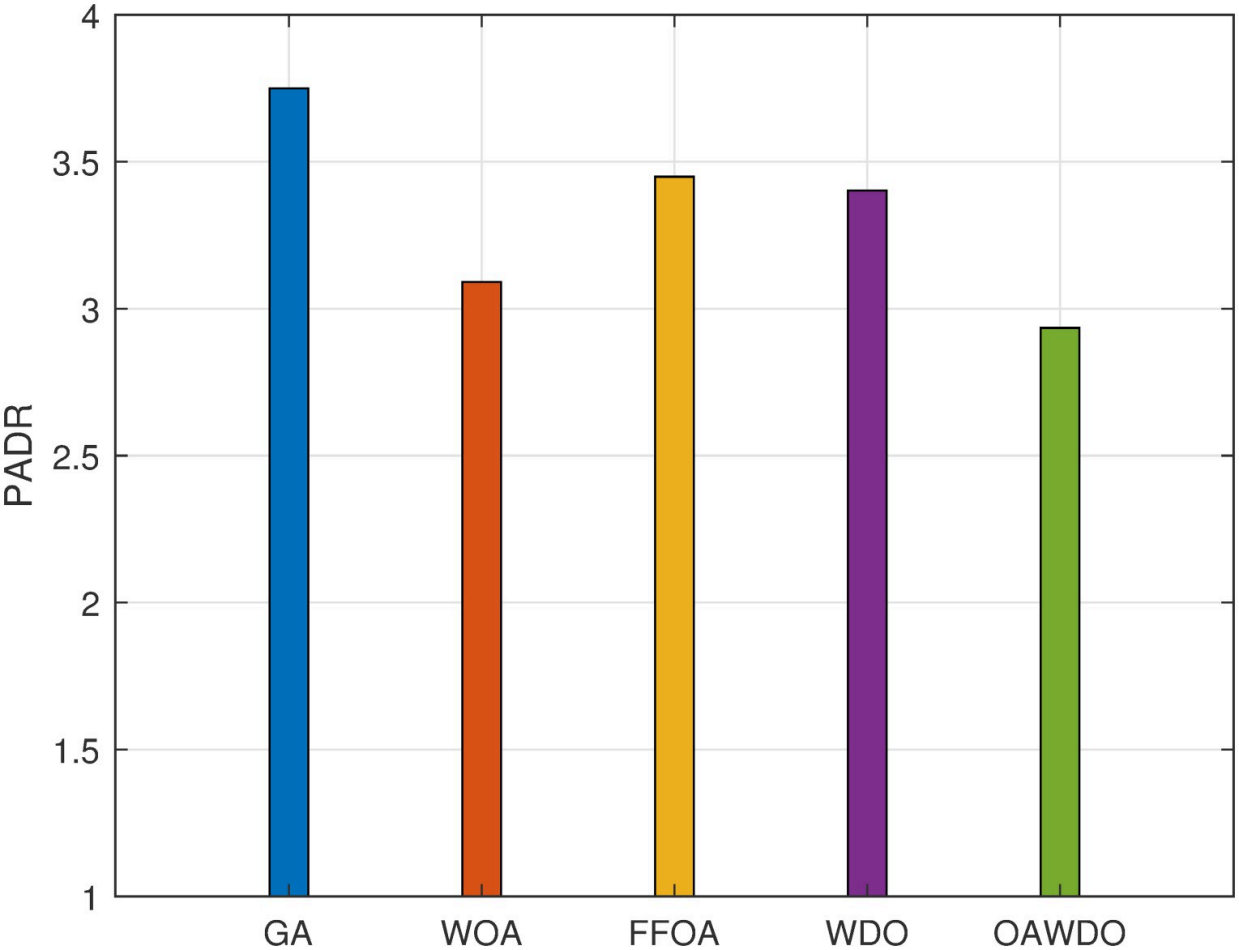
Case 2: PADR with load scheduling.

The assessment of PADR for load scheduling using SPVE and storage batteries is visualized in Fig 14. The PADR for the GA, WOA, FFOA, WDO, and OAWDO are 3.8, 3.1, 3.49, 3.3, and 2.9, respectively.

The OAWDO method demonstrates superior performance compared to alternative algorithms such as GA, WOA, FFOA, and WDO in minimizing the PADR. Specifically, it achieves a reduction of 23.68%, which is significantly higher compared to the reductions achieved by GA, WOA, FFOA, and WDO, which are 18.42%, 8.15%, and 13.15%, respectively. This notable improvement can be attributed to OAWDO’s ability to evenly distribute the load between times of high and low demand, effectively mitigating the formation of rebound peaks. Conversely, other algorithms tend to redistribute the load unevenly, leading to the formation of rebound peaks and subsequent reliability issues for utility providers. By uniformly distributing the load, OAWDO successfully addresses this issue, which results in a substantial reduction in PADR. This reduction in PADR is not only beneficial for utility providers but also advantageous for users, as it helps optimize energy consumption and reduces costs during peak hours. Overall, the findings underscore the efficiency and effectiveness of the OAWDO algorithm in load shifting.

**Fig 15:**
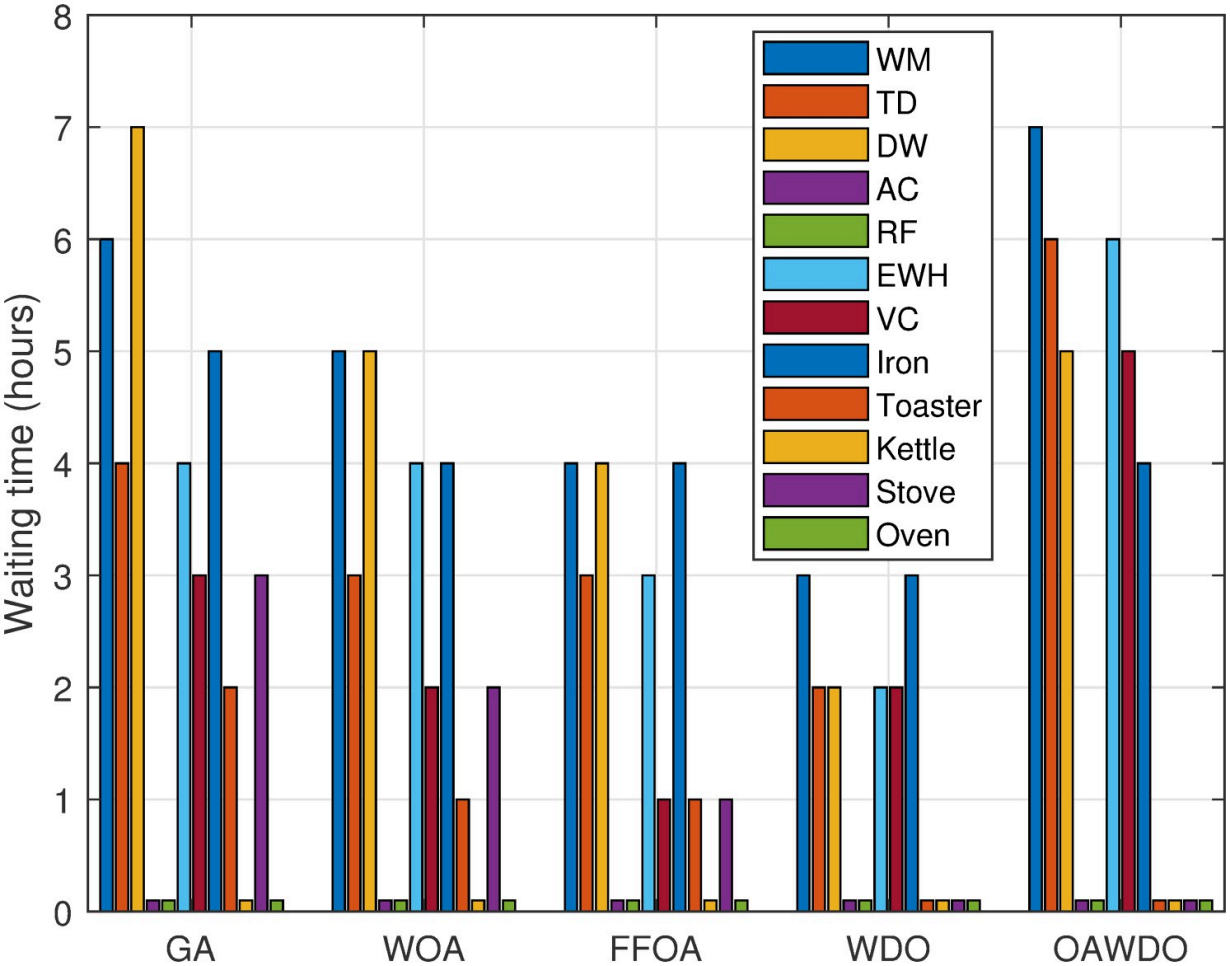
Case 2: Consumer comfort in terms of waiting time with scheduling.

The schedules generated by OAWDO are compared with schedules produced by other algorithms to evaluate user dissatisfaction in terms of delays encountered by consumers. Consumer comfort, specifically regarding waiting time, is assessed for the OAWDO and comparative algorithms, as illustrated in Fig 15. In the GA, the returned schedule for devices such as WM, TD, DW, EWH, VC, iron, toaster, and stove experience average delays of 6, 4, 7, 0, 4, 3, 5, 2, 3, and 0 hours, respectively. In contrast, appliances like RF, AC, kettle, and oven encounter zero delays. The schedule created by the WOA algorithm results in a mean delay of 5, 3, 5, 4, 2, 4, 1, and 2 hours for appliances such as WM, TD, DW, EWH, VC, iron, toaster, and stove, respectively. Conversely, RF, AC, kettle, and oven experience zero delays. The schedule created by the FFOA algorithm results in average delays of 4, 3, 4, 3, 1, 4, 1, and 1 hours for appliances like WM, TD, DW, EWH, VC, iron, toaster, and stove, respectively. Conversely, RF, AC, kettle, and oven experience zero delays. In the OAWDO-created schedule, average delays of 7, 6, 6, 5, 5, and 4 hours are observed for appliances like WM, TD, EWH, DW, VC, and iron, respectively. Appliances like RF, AC, toasters, kettles, stoves, and ovens experience zero delays. The evaluation demonstrates that the created OAWDO algorithm surpasses other algorithms in terms of average waiting time, hence improving consumer satisfaction. The OAWDO algorithm delays the operation of mostly shiftable appliances and does not distribute delays to appliances whose nature is either power flexible or critical.

The comparison of the proposed framework using OAWDO with other frameworks is displayed in Table 2. The assessment encompasses factors such as electricity bills, PADR, and reduction in consumer discomfort. The findings highlight the superiority of the suggested framework compared to alternative ones in efficiently accomplishing the given objectives.

**Table 2:** Performance of analysis across various objectives includes evaluating electricity expenses, PADR, and consumer dissatisfaction.

Technique(s)	Electricity expenses (%)	PADR (%)	Comfort (%)	Computational complexity (sec)
WOA	8.27	18.42	2.19	146
FFOA	9.77	8.15	1.78	123
WDO	11.27	13.15	1.29	105
OAWDO	17.66	23.68	2.87	101

## Conclusions

This study presents a novel energy optimization framework tailored for flexible load optimization with RTP-IBT, integrating SPVE and storage batteries. The introduced OAWDO algorithm addresses the energy optimization problem in two cases: both with and without SPVE and storage batteries integration, with the overarching goal of reducing electricity expenses and PADR while maintaining consumer satisfaction. The study validates the proposed model through simulations and compares it with alternative frameworks utilizing GA, WOA, FFOA, and WDO schemes. The findings highlight the effectiveness of the OAWDO algorithm, demonstrating significant improvements in key metrics. Notably, the algorithm achieves a substantial reduction in electricity bills (17.66%), PADR (23.68%), and average waiting time (2.87). These findings highlight the capability of the proposed model to improve the efficiency of load scheduling, offering promising benefits for both consumers and utility companies within smart power grids.

## Abbreviations

λ1, λ2: Scale factors
ℜ: Peak-to-Average Demand Ratio
∂_*pv*_: SPVE panel efficiency
$p_{SB}^{ch}\left(t\right)$: Charging power of the battery
$p_{SB}^{dch}\left(t\right)$: Discharging power of the battery
$\gamma_{SB}^{PV}$: Net electricity bill with SPVE and batteries
$p_{t}^{g}$: Power obtained from the grid
$p_{t}^{PV}$: Power obtained from SPVE
$p_{t}^{l}$: Power consumed by loads
$p_{SB}^{ch}\left(t\right)$: Charging power of the storage battery
$p_{SB}^{dch}\left(t\right)$: Discharging power of the storage battery
$E_{SB}^{\text{min}},E_{SB}^{\text{max}}$: Lower and upper bounds of energy stored in the battery
$\varepsilon_{T}^{bsch},\varepsilon_{T}^{asch}$: Energy consumption before and after scheduling
$T_{i}^{l,bsch},T_{i}^{l,asch}$: Duration length of appliance operation before and after scheduling
$X_{t}^{i,bsch},X_{t}^{i,asch}$: Appliance operation status before and after scheduling
*γ*: Net electricity bill
*η*_*ch*_, *η*_*dch*_: Charging and discharging efficiencies
*θ*1, *θ*2: Shape factors
*κ*: Waiting time
*φ*(*t*): RTP-IBT signal
*ω*: Weigh factor
*A*_*pv*_: Solar panel area
*E*_*SB*_(*t*): Energy stored in the battery
*f*: Objective function
*P*_*pv*_: Generated output power
*rad*(*t*): Irradiation
*SOC*(*t*): State of charge
*SOC*^min^, *SOC*^max^: Maximum and minimum upper bounds of state of charge
*Tp*(*t*): Temperature
*w*_1_, *w*_2_, *w*_3_: Non-negative weights for the electricity bill, PADR, and user discomfort
CPP: Critical Peak Pricing
DAP: Day-Ahead Pricing
DR: Demand Response
LSC: Load Scheduling Controller
OAWDO: Optimal Adaptive Wind-Driven Optimization
PADR: Peak-to-Average Demand Ratio
RTP: Real-Time Pricing
SPVE: Solar Photovoltaic Energy
ToU: Time of Use
WDO: Wind-Driven Optimization
